# Health literacy in pregnant women facing prenatal screening may explain their intention to use a patient decision aid: a short report

**DOI:** 10.1186/s13104-016-2141-0

**Published:** 2016-07-11

**Authors:** Agathe Delanoë, Johanie Lépine, Maria Esther Leiva Portocarrero, Hubert Robitaille, Stéphane Turcotte, Isabelle Lévesque, Brenda J. Wilson, Anik M. C. Giguère, France Légaré

**Affiliations:** Canada Research Chair in Shared Decision Making and Knowledge Translation, Public Health and Practice-Changing Research Group, Centre Hospitalier Universitaire de Québec Research Centre, Hôpital St-François d’Assise, 10 rue Espinay, D6-737, Quebec City, QC G1L 3L5 Canada; Obstetrics and Gynecology Department, Faculty of Medicine, Université Laval, 1050, avenue de la Médecine, Quebec City, QC Canada; Department of Epidemiology and Community Medicine, Faculty of Medicine, University of Ottawa, Roger Guindon Hall, 451 Smyth Road, Ottawa, ON Canada; Quebec Centre of Excellence on Aging, CHU de Québec Research Centre, 1050, chemin Sainte-Foy, Quebec City, QC Canada; Department of Family Medicine and Emergency Medicine, Faculty of Medicine, Université Laval, 1050, avenue de la Médecine, Quebec City, QC Canada

**Keywords:** Patient decision aid, Shared decision making, Patient involvement, Health literacy, Screening and diagnostic tests, Down syndrome

## Abstract

**Background:**

It has been suggested that health literacy may impact the use of decision aids (DAs) among patients facing difficult decisions. Embedded in the pilot test of a questionnaire, this study aimed to measure the association between health literacy and pregnant women’s intention to use a DA to decide about prenatal screening. We recruited a convenience sample of 45 pregnant women in three clinical sites (family practice teaching unit, birthing center and obstetrical ambulatory care clinic). We asked participating women to complete a self-administered questionnaire assessing their intention to use a DA to decide about prenatal screening and assessed their health literacy levels using one subjective and two objective scales.

**Results:**

Two of the three scales discriminated between levels of health literacy (three numeracy questions and three health literacy questions). We found a positive correlation between pregnant women’s intention to use a DA and subjective health literacy (Spearman coefficient, Rho 0.32, *P* = 0.04) but not objective health literacy (Spearman coefficient, Rho 0.07, *P* = 0.65). Hence subjective health literacy may affect the intention to use a DA among pregnant women facing a decision about prenatal screening.

**Conclusion:**

Special attention should be given to pregnant women with lower health literacy levels to increase their intention to use a DA and ensure that every pregnant women can give informed and value-based consent to prenatal screening.

**Electronic supplementary material:**

The online version of this article (doi:10.1186/s13104-016-2141-0) contains supplementary material, which is available to authorized users.

## Background

Prenatal screening is routinely offered in many industrialized countries [1]. In the province of Quebec, the public healthcare system freely offers the serum integrated test to all pregnant women on a voluntary basis in routine prenatal care consultations. It involves two blood tests taken between the 10th and 16th weeks of pregnancy. The new non-invasive prenatal test (NIPT) provides earlier and more accurate results for DS but is mainly only offered by private companies. To the best of our knowledge, DAs are not used in the context of routine prenatal screening offered in the public system [2]. Indeed, we recently conducted an environmental scan of all DAs for prenatal screening, and found only two in Canada, and none of the 20 found worldwide met the International Patient Decision Aids Standards [3]. While prenatal screening results may decrease pregnant women’s uncertainty, there are risks of false positive or false negative results. Furthermore, women identified at high risk have then to decide whether or not to undergo amniocentesis, an invasive test that entails a risk of pregnancy loss. Depending on the results of this diagnostic test, the woman then has to decide between having an abortion or preparing for a child with special needs. This in turn may involve a major challenge to personal life values and a change to one’s hopes for the future. Thus a decision about prenatal screening may seem banal in itself, but can be just the first in a series of decisions of increasing sensitivity and difficulty. DAs are tools that foster shared decision-making by supporting patients and their health professionals as they attempt to agree on a decision point, discuss evidence and clarify what is most important for the patient [4–7]. Although DAs have been shown to produce favorable decision outcomes for patients [8, 9], there is a consensus that they have not been routinely implemented in care in general [5]. This is due to various barriers, including lack of training in how to use decision support, a lack of trust in or agreement with the content of the DA, or the belief among health professionals that patients do not want decisional responsibility when facing difficult diagnoses [5]. This also holds true for the implementation of decision aids in prenatal care [10]. For example, two recently completed studies on factors influencing the implementation of decision aids in prenatal care suggested that the main factors that influence the use of a DA by health professionals were a positive appraisal of the DA, its availability in the office, and colleagues’ approval [11]; and that main factors influencing pregnant women’s use of a DA were the opinion of her partner, the presentation of the DA by the health professional and a discussion, and having never before encountered a DA [12]. Many factors are thus thought to affect their effective implementation [13] and previous research has hypothesized that patient health literacy could be one such factor [14].

Health literacy is defined as all the “cognitive and social skills which determine the motivation and ability of individuals to gain access to, understand and use information in ways which promote and maintain good health [15].” It thus includes dimensions such as self-confidence and social networks as well as literacy and numeracy. DAs help patients interact with their healthcare professionals to understand evidence and construct informed preferences, but using them may also involve these dimensions of health literacy [14–16]. Many studies have reported that health literacy influences patients’ attitudes toward shared decision-making [17–21] as it is linked to their understanding and preferences when making health-related decisions [22–24]. However, to the best of our knowledge, no study has yet assessed the relationship between health literacy and the intention to use DAs among pregnant women facing prenatal screening. Any correlation would underline the importance of designing decision aids that maximize this intention among women of every health literacy level, ultimately enabling them to make an informed and value-based decision. Consequently, we sought to explore the association between health literacy and pregnant women’s intention to use a DA to decide about prenatal screening for DS.

## Methods

### Study design and participants

**Fig. 1:**
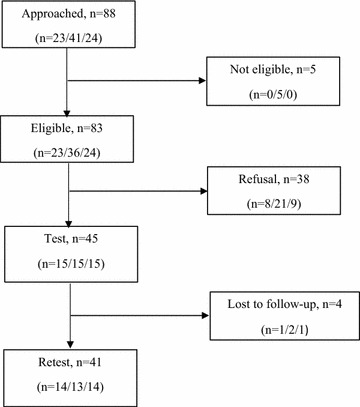
Flow of participants. Recruitment (approached, eligible/not eligible and agree/refuse to participate) and data collection (test, lost to follow-up/retest). *Numbers* in parenthesis are presented in this order: pregnant women receiving care from family physicians, midwives or obstetricians–gynecologists

This study was embedded in the pilot test of a questionnaire (2-week test–retest) aiming to assess the theory-based factors influencing the use of a DA to decide about prenatal screening. Between March and April 2015, we targeted a convenience sample of 45 pregnant women in three clinical sites (family practice teaching unit, birthing center and obstetrical clinic) in Quebec City, Canada. Pregnant women were monitored by either family physicians, midwives or obstetrician–gynecologists, respectively. Inclusion criteria were (1) minimum age of 18 years; (2) being in the second trimester of pregnancy; and (3) pregnancy not classified at high-risk of complications, excluding DS risk (i.e. preeclampsia, gestational diabetes, and multiple pregnancy). Women were approached consecutively in the waiting room, before and after their follow-up appointments. Of the 88 pregnant women invited to participate, 83 were found to be eligible and, of these, 45 (54 %) agreed to participate in the study (Fig. 1). The study was conducted in French and all participating women provided informed consent.

### Data collection

Based on previous work assessing factors influencing health related behavior change [25, 26], we observed that in order for respondents to understand the nature of the behavior being studied, it is most helpful to give them a vicarious experience of the behavior of interest [27]. We therefore ensured that all of them watched a 10-minute video that depicted a prenatal care follow-up during which a pregnant woman, her partner and a health professional used a DA to decide about prenatal screening for DS. The production of this video followed a validated process successfully used elsewhere that includes rigorous peer-review validation of its accurate and true-to-life depiction of the behavior [28]. After watching the video, participants filled out the first self-administered questionnaire (test). They completed the questionnaire online two weeks later (retest). We assessed pregnant women’s intention to use a DA to decide about prenatal screening using two closed-ended questions (scored on a 5-point Likert scale) based on the Theory of Planned Behavior [29]. Questions were: (1) I intend to use a DA…; (2) I would use a DA…. Response range was from Strongly disagree to Strongly agree. After consulting with experts in the field [30–33] and reviewing multiple systematic reviews [34–38], we chose to use one subjective scale [39] and two objective scales [40, 41]. Both subjective and objective assessments of health literacy are important. While objective scales measure competencies, subjective scales measure the perception of competencies, and reduce participant burden [42, 43].

To decrease participants’ burden, we split the administration of the three scales between the test and retest phase. During the test, we used two scales: (1) the three numeracy questions (3NQ), a self-administered three-question scale that objectively measures health numeracy (three items asking respondents to convert proportions, probabilities and percentages, correct answers range 0–3, see Additional file 1: Appendix S1) [41] and (2) the newest vital sign (NVS), a six-item scale that also objectively measures health literacy but is orally administered by a research assistant (six orally administered questions that assess understanding of an ice cream label, see Additional file 1: Appendix S2) [40]. During the retest, we used the three health literacy questions (3HLQ), a self-administered three-question scale that subjectively measures health literacy (three items about self-confidence, social support and learning problems, scored on a five-point Likert scale: range 0–4, final score range 0–12, see Additional file 1: Appendix S3) [39]. Sociodemographic characteristics were also collected.

### Data analysis

We used simple descriptive statistics to summarize participant demographics, intention levels and health literacy scores. The internal consistency of the intention construct was first verified using Spearman’s correlations for both test and retest questionnaires. Intention scores were then computed using the mean of the two items in both test and retest. Reliability of the intention construct was confirmed by performing a Wilcoxon test. The mean score was calculated for each health literacy scale (ranges: 3NQ, 0–3; NVS, 0–6; 3HLQ, 0–12). We computed the discriminating capacity of each scale by plotting the number of pregnant women against the number of correct answers for the objective scales (3NQ and NVS), and against the total score for the subjective scale (3HLQ). Lack of variability in the scale indicated an absence of discriminating capacity. For each health literacy scale and intention item, a higher score indicates higher literacy or stronger intention. We explored associations between health literacy and intention to use a DA using Spearman’s correlations only with health literacy scales that showed discriminating capacity. We did not impute any data since there were no missing data except for data on pregnancy and education. Data analyses were performed using SAS 9.4 software.

## Results

### Participants’ characteristics

The women’s characteristics are detailed in Table 1.

**Table 1:** Participant characteristics

	Family physicians n = 15 (%)	Midwives n = 15 (%)	Obst. Gyn. n = 15 (%)	Total n = 45 (%)
Age				
Mean (median; range)	30.9 (31.0; 20–42)	31.4 (33.0; 25-35)	31 (31.0; 26–36)	31.1 (31.0; 20–42)
Marital status				
Single	0 (0 %)	3 (20 %)	0 (0 %)	3 (7 %)
Not single	15 (100 %)	12 (80 %)	15 (100 %)	42 (93 %)
Education^a^				
High school or less	4 (29 %)	1 (7 %)	2 (13 %)	7 (16 %)
College (years 12 and 13)	4 (29 %)	5 (33 %)	4 (27 %)	13 (30 %)
University	6 (42 %)	9 (60 %)	9 (60 %)	24 (54 %)
Pregnancy^a^				
1st	2 (14 %)	2 (13 %)	6 (40 %)	10 (23 %)
2nd	8 (57 %)	3 (20 %)	5 (33 %)	16 (36 %)
≥3rd	4 (29 %)	10 (67 %)	4 (27 %)	18 (41 %)

^a^One missing data among women followed by family physicians

### Intention and health literacy scores

**Fig. 2:**
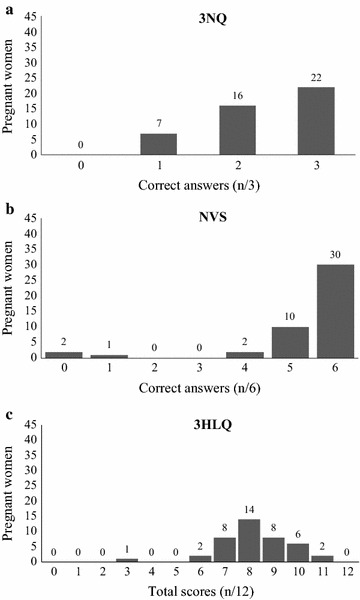
Health literacy scales distribution. **a** Distribution of the sample against the number of correct answers for the 3NQ scale. **b** Distribution of the sample against the number of correct answers for the NVS scale. **c** Distribution of the sample against the total score for the 3HLQ scale

Pregnant women’s intention levels showed a median score of 4.5 at the test and 4.0 at the retest (range from 1 to 5, Table 2). Intention levels were not significantly different between the test and retest (*P* > 0.05). On the 3NQ scale the median score was 2 out of 3 (Table 2), and 49 % of the sample obtained the maximum score (3/3, n = 22/45, Fig. 2a). The median score on the NVS was 6 out of 6 (Table 2), and 67 % of the sample correctly answered all questions (6/6, n = 30/45, Fig. 2b). Furthermore, 89 % of pregnant women scored 5 or 6 out of 6 (n = 40/45, Fig. 2b). Finally, the median score on the 3HLQ was 8 out of 12 (Table 2). The variable was negatively skewed but showed variability around the median score (Fig. 2c).

**Table 2:** Pregnant women’s intention and health literacy levels

	Intention level^a^		Health literacy level		
	Test^b^	Retest^c^	Test 3NQ	Test NVS	Retest 3HLQ
Median	4.5/5	4.0/5	2/3	6/6	8/12^d^
Mean ± SD	4.3 ± 0.9	4.1 ± 0.9	2.3 ± 0.7	5.3 ± 1.6	8.2 ± 1.6

^a^Reliability: *P* > 0.05 (Wilcoxon test), meaning that no statistical difference was found between the two measures of intention

^b^Internal consistency: 0.7 (Spearman coefficient, Rho, *P* < *0.0001*)

^c^Internal consistency: 0.9 (Spearman coefficient, Rho, *P* < *0.0001*)

^d^Range from 0 to 12, with 0 indicating low health literacy, and 12 high. Scoring for this scale has been reversed from its original position [39] so that health literacy ranges for all scales are easier to compare

### Association between pregnant women’s intention and health literacy

We observed no significant correlation between pregnant women’s intention to use a DA to decide about DS screening and their score on the objective health numeracy scale (3NQ, Spearman coefficient, Rho 0.07, *P* = 0.65). Taking into account the weak discriminating power of the NVS, no further analysis was performed with this scale. However, we observed a significant and positive association between subjective health literacy and the intention to use a DA for prenatal screening (3HLQ, Spearman coefficient, Rho 0.32, *P* = 0.04).

## Discussion

We sought to explore the association between health literacy and pregnant women’s intention to use a DA for prenatal screening. In general, health literacy scores in pregnant women were high. A positive association was observed when health literacy was assessed subjectively (i.e. the higher the self-perceived health literacy, the higher the intention), but not when assessed objectively. To the best of our knowledge, this study is among the first to document the relationship between pregnant women’s intention to use a DA and health literacy. Both the 3NQ and the 3HLQ scales had sufficient discriminating capacity to be used in further studies in pregnant women without high risk pregnancy. In contrast, the NVS scale was not considered discriminative enough to be used in this population. These results lead us to make two main points.

First, it was only when women’s health literacy was evaluated subjectively that a relationship was observed between health literacy and their intention to use a DA. Thus, when deciding about DS screening, pregnant women’s own perceptions of their self-confidence, reading comprehension and social support may influence their intention to use a DA. This confirms the idea that objective outcomes in healthcare communications interventions do not always match outcomes perceived by the target population [44, 45]. Therefore, training health professionals in decision support that increases pregnant women’s self-confidence or considers their social support network may increase their intention to use a DA about prenatal screening.

Second, we administered three scales, but in this specific context only two discriminated among health literacy levels. Overall, the health literacy level was high, and most participants had a high education level. As education is an important predictor of health literacy, this could explain the relatively weak discriminating capacity of the NVS scale [46]. However, our study is among the first to provide evidence about health literacy scales in a French-speaking population and thus will help build knowledge in this area.

Our results need to be interpreted with caution, as we used a convenience sample in three clinical sites of the same city, limiting generalization. With a larger sample, it would also be interesting to evaluate if older pregnant women, who are at higher risk of carrying a fetus with DS, have a higher intention to use a DA. Only 51 % of the invited women agreed to participate and, as we did not know if their characteristics were similar to those of the women who declined, the study sample might not be representative of the population of interest. However, our results may be a useful basis for future systematic reviews or larger studies in this area. It is also possible that the video mediated intent; however, we felt it was more important to ensure that respondents understood the nature of the behavior being studied than to avoid any risk of mediated intent by not using a video at all. This pilot study enabled us to validate a questionnaire about pregnant women’s intention to use a DA for DS screening and to select relevant health literacy scales for that population, providing preliminary data before a broader survey is conducted across Quebec province. Furthermore, our study provides new data on three health literacy scales.

## Conclusion

This study showed a modest but significant association between health literacy and pregnant women’s intention to use a DA to decide about prenatal screening, and the difference between outcomes obtained from subjective assessments and those obtained from objective assessments. Special attention should be given to pregnant women with lower health literacy levels to increase their intention to use a DA and ensure that every pregnant women can give informed and value-based consent to prenatal screening. Once DAs are implemented on a wider scale, it will be possible to evaluate if this intent results in action.

## Additional file

10.1186/s13104-016-2141-0 Additional appendices.

## Abbreviations

DA: decision aid
DS: down syndrome
3NQ: three numeracy questions (objective numeracy scale)
NVS: newest vital sign (objective health literacy scale)
3HLQ: three health literacy questions (subjective health literacy scale)
