# Supplementary material for: Health literacy in pregnant women facing prenatal screening may explain their intention to use a patient decision aid: a short report
Source: BMC Res Notes. 2016 Jul 11;9:339. doi: 10.1186/s13104-016-2141-0 (PMC4940686; doi:10.1186/s13104-016-2141-0)
Supplement: Supplementary file 1 — 10.1186/s13104-016-2141-0 Additional appendices. [file 13104_2016_2141_MOESM1_ESM.doc]

Appendices

1. **3NQ (adapted from Schwartz et al., 1997)**
2. A person who takes medication “X” has 1% chance of having an allergic reaction. If 1000 people take medication “X”, how many will have an allergic reaction?

……………. person(s) out of 1000

1. A person who takes medication “Y” has 1 chance in 1000 of having an allergic reaction. What percentage of people who take medication “Y” will have an allergic reaction?

…………….%

1. Imagine that you toss a coin 1000 times for heads or tails. At your best guess, how many times out of 1000 will you get heads?

…………… times out of 1000

1. **NVS (Weiss et al., 2005)**


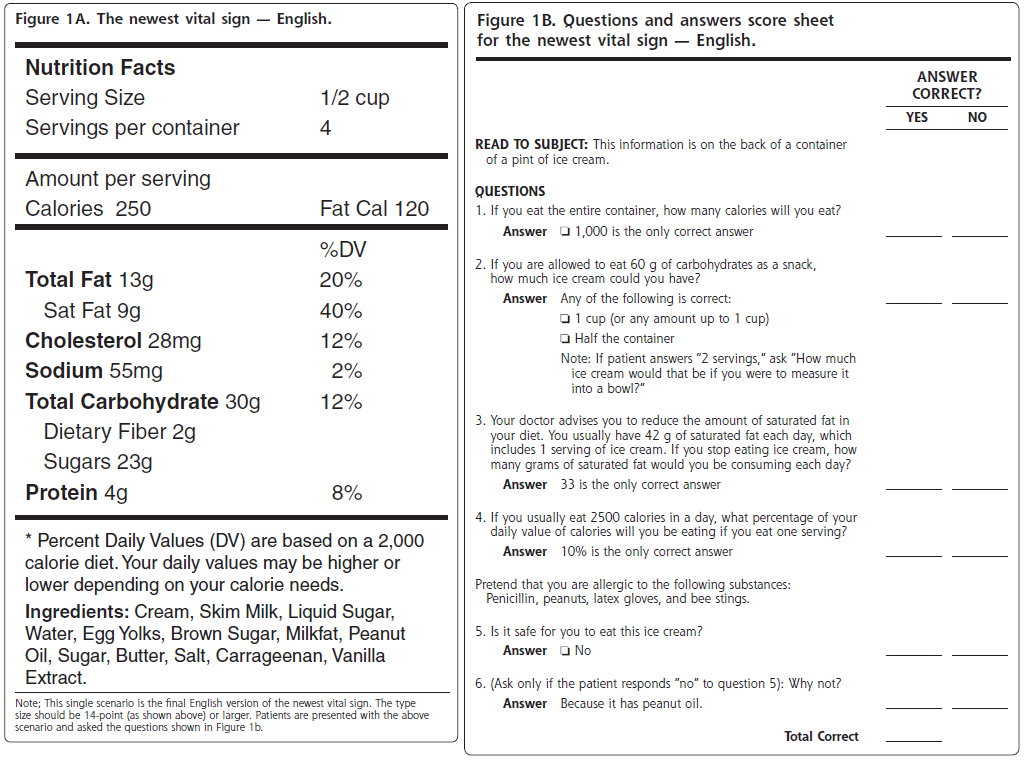


1. **/////3HLQ (Chew et al., 2004)**
2. How confident are you filling forms by yourself?

(*Not at all confident, a little bit confident, somewhat confident, quite a bit confident, extremely confident*)

1. How often do you have someone help you read hospital materials?

(*Never, occasionally, sometimes, often, always*)

1. How often do you have problems learning about your medical conditions because of difficulty reading hospital materials?

(*Never, occasionally, sometimes, often, always*)
